# The expression and construction of engineering *Escherichia coli* producing humanized AluY RNAs

**DOI:** 10.1186/s12934-017-0800-z

**Published:** 2017-10-30

**Authors:** Chao Liu, Yuehua Zhao, Shuxian Yin, Shufeng Liu, Huanling Zhang, Xiufang Wang, Zhanjun Lv

**Affiliations:** 1 Department of Genetics, Hebei Key Lab of Laboratory Animal, Hebei Medical University, Shijiazhuang, 050017 Hebei Province China; 2 School of Stomatology, Hebei Medical University, Shijiazhuang, 050017 Hebei Province China

**Keywords:** Genetically engineered RNA, AluY RNAs, IPTG induction, BL21 (DE3)

## Abstract

**Background:**

Exogenous RNAs can specifically up-regulate or down-regulate gene expression after they enter into cells. Alu RNAs are the main constituent of human transcriptome and participate in gene expression regulation. AluY elements belong to a subfamily of Alus and are the youngest Alus. In this paper, we established the technology method of preparing genetically engineered humanized AluY RNAs (AluY RNAs) from *Escherichia coli* (*E. coli*) strains. This technology method also can be used to prepare other genetically engineered humanized RNAs that can be used for cytology experiments.

**Results:**

Different copies of human AluY elements were inserted into pET-28α plasmid (pET) to construct pET-AluY plasmids that were transformed into *BM*BL21-DE3 (DE3) *E. coli*. Isopropylthio-β-d-galactoside (IPTG) induction inhibited transformed bacterial growth after DE3 *E. coli* were transformed by pET-AluY × 8 plasmid (8 copies of AluYs were inserted into pET); northern blotting was used to detect the amount of AluY RNAs after 2, 4, 6, 8, 10, 12, 14 and 16 h inducing with IPTG. The results showed that the amount of AluY RNAs was the highest at 4 h; 1, 2, 4, 8 or 14 copies of AluY elements were inserted into the pET to construct pET-AluY plasmids that were transformed into DE3 bacteria, the northern blotting results showed that AluY RNAs production amount increased with the increase of AluY copy number; pET-AluY × 8 DE3 bacteria did not produce AluY RNAs without IPTG induction, AluY RNA production kept similar when inducing by 0.1–0.4 mg/ml IPTG induction, however, AluY RNA production slightly decreased if deviating from the above concentration range; pET-AluY × 8 DE3 bacteria were cultured at 34, 37 or 40 °C and the results showed that AluY RNA production was the highest under 37 °C cultivation; pET-AluY × 8 plasmid was transformed into three kinds of BL21 bacteria, including DE3, *BM*BL21-DE3-pLysS (pLysS) and Trans BL 21 (TransBL), the results showed that AluY RNA production was the highest when using DE3 bacteria.

**Conclusions:**

The optimal conditions of producing AluY RNAs were: a kind of host bacteria of DE3, an engineering bacteria concentration of OD_600_ 1.0, an IPTG concentration of 0.2 mg/ml, a culturing temperature of 37 °C and a culturing time of 4 h. Pure AluY RNAs occupied 15.8% of extractive total RNAs and the mean yield of pure AluY RNAs in 100 ml bacteria solution was 0.46 mg.

## Background

RNAs as important factors of regulating gene expression increasingly arouse people’s attention [1]. It has been shown that RNAs, which include microRNA [2, 3], small activating RNA [4, 5], non-coding RNA [6, 7] etc., can up-regulate or down-regulate gene expression. RNAs in cells can transcribe from DNAs (genome DNA or transfected plasmids) or import from extracellular RNAs [5, 8]. The methods of acquiring extracellular RNAs include RNA synthesis using RNA synthesizer [9], in vitro transcription [10] and extraction from cells [11]. Alu elements are most important non-coding sequences and comprise about 10% of the human genome [12, 13]. It has been shown that Alu sequences affect gene expression [14, 15]. Alu elements are abundant short interspersed nuclear elements in primate genomes and are present in more than one million copies in human genome. Alu sequences were initially considered as parasites of the human genome that had no major effect on genomic stability and gene expression [16]. However, as research continues, Alu functions in gene regulation and expression networks are gradually being found. Alu elements are closely related to human disease. Alu elements insertions, deletion and recombination occur in human populations, and have been responsible for several instances of genetic disease and cancers, in addition, may affect human aging and also play important roles in human diversity [17]. Although Alu sequences functional effects are poorly understood at present, it has been speculated that Alu elements participate in gene expression regulation, gene rearrangement, CpG methylation, hnRNA alternative splicing, binding with transcription factors and hormone etc. [18, 19]. Alu elements were split in three main subfamilies including AluJB, AluS and AluY. The AluY elements are the youngest of the three and have the greatest disposition to move along the human genome [20]. In this paper, the preparation technology of genetically engineered humanized AluY RNAs (AluY RNAs) was established. This technology method also can be used to prepare other genetically engineered humanized RNAs that can be used for cytology experiments.

## Results

### The effects of IPTG induction on the growth of the bacteria and yield of AluY RNAs producing from pET-AluY × 8 DE3 bacteria

**Fig. 1:**
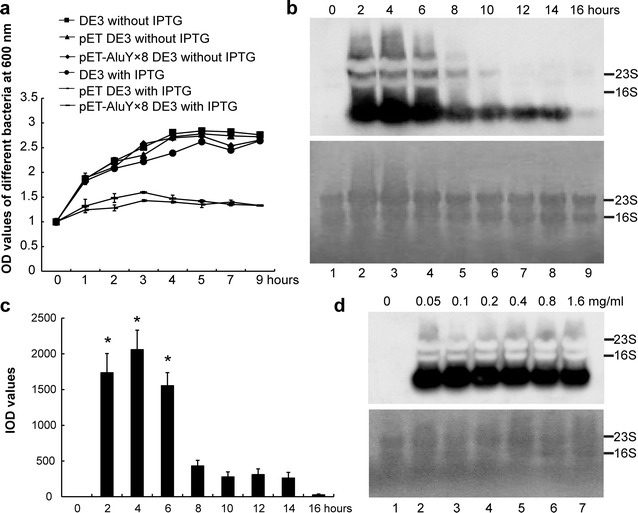
The effects of IPTG induction on the bacterial growth and yield of AluY RNAs producing from pET-AluY × 8 DE3 bacteria. **a** Effects of IPTG induction on the bacterial growth; **b** effects of IPTG induction time on the yield of AluY RNAs. The top panel shows the results of northern blotting after different time induced by IPTG; the bottom panel shows the loading amount of RNAs in each lane dyed by methylene blue; **c** IOD values of each lane of the northern blotting results (means of three independent experiments) after different time induced by IPTG. “*”, the yield of AluY RNAs when IPTG induction for 2–6 h was significantly higher than that of other induction times (p < 0.01). **d** Effects of IPTG concentrations on yield of AluY RNAs. The top panel shows the northern detection results after 4 h induction by different concentrations of IPTG; the bottom panel shows the loading amount of RNAs in each lane dyed by methylene blue

pET-AluY × 8 DE3 (DE3 bacteria transformed by pET-AluY × 8 vector (8 copies of AluYs were inserted into pET-28ɑ)), pET DE3 and DE3 were cultured using LB-kanamycin medium, respectively. pET-28ɑ was abbreviated as pET in this paper. The bacteria were adjusted to OD_600_ 1.0, induced with isopropyl-β-d-thiogalactoside (IPTG) or without IPTG, cultured another 1, 2, 3, 4, 5, 7 or 9 h, a portion of bacteria were used to assess their OD. Figure 1a shows that the growth of all types of bacteria reached plateau at OD 2.6 when without IPTG induction, while the growth of pET-AluY × 8 DE3 and pET DE3 bacteria reached plateau at OD 1.3 with IPTG induction, which illustrates that IPTG induction inhibited the growth of the two types of bacteria, IPTG induction only had almost no effect on the growth of DE3.

pET-AluY × 8 DE3 bacteria were cultured at 37 °C to OD_600_ 1.0, induced by IPTG (0.2 mg/ml) for 2, 4, 6, 8, 10, 12, 14 or 16 h. AluY RNAs were extracted using SDS-hot phenol method [11] as described in “Methods” and detected using northern blotting. The results showed that AluY RNA expression amount reached peak at 2–6 h, decreased at 8–14 h, and then obviously decreased at 16 h (Fig. 1b, the top panel). The bottom panel of Fig. 1b shows the loading amount of RNAs dyed by methylene blue in each panel. Gel-Pro-analyzer software was used to analyze the integral optical density (IOD) value of each lane of the northern blotting results (Fig. 1c). The growth process of DE3 bacteria contains lag phase, exponential phase, stationary phase and death phase. In this paper we found that IPTG induction inhibited the growth of the pET-AluY × 8 DE3 bacteria, but the growth process of pET-AluY × 8 DE3 bacteria still exerted lag phase, exponential phase, stationary phase and death phase. We analyzed the reason that AluY RNA expression amount changed with incubation time: (1) AluY RNA expression amount was effected by growth behavior of DE3 bacteria with incubation time; (2) during exponential phase, AluY RNA accumulation increased since DE3 bacteria were in good growth condition; during stationary phase, the degradation of AluY RNAs kept the similar with the production of AluY RNAs; during the death phase, AluY RNA production was lesser than the degradation of AluY RNAs.

pET-AluY × 8 DE3 bacteria did not produce AluY RNAs without IPTG induction and produced similar amount AluY RNAs with 0.1–0.4 mg/ml IPTG induction. AluY RNA yield slightly decreased when induction with other concentration IPTG (Fig. 1d).

### The effects of inserted copy number of AluY elements on the yield of AluY RNAs

**Fig. 2:**
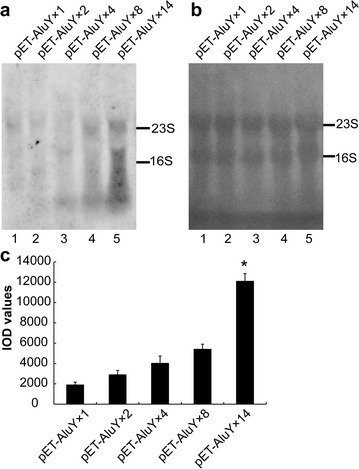
Effect of copy number of AluYs on AluY RNA production. **a** The results of northern detection. 1, 2, 4, 8 or 14 copies of AluY elements were inserted into the pET plasmid to construct pET-AluY plasmids that were transformed into DE3 bacteria that were induced with IPTG (final concentration 0.2 mg/ml) at 37 °C. The northern blot results showed that AluY RNA production amount increased with the increase of AluY copy number; **b** dyeing result of methylene blue; **c** IOD values of each lane of the northern blotting results (means of three independent experiments. “*”, the AluY RNAs yield of pET-AluY × 14 plasmid was significantly higher than that of other plasmids (p < 0.01)

pET-AluY × 1 DE3, pET-AluY × 2 DE3, pET-AluY × 4 DE3, pET-AluY × 8 DE3 and pET-AluY × 14 DE3 bacteria were induced for 4 h when OD value was 1.0. AluY RNAs were extracted and assessed using northern blotting. Figure 2a shows that the yield of AluY RNAs increased with increasing the copy number of AluY elements. The yield of pET-AluY × 14 (Fig. 2a, line 5) was 6.4-fold of pET-AluY × 1 (Fig. 2a, line 1). Figure 2b shows the loading amount of RNAs dyed using methylene blue in each lane was similar. Figure 2c shows the IOD value of each lane of the northern blotting results (means of three independent experiments). The reason that the pET-AluY × 14 vector produced the most AluY RNAs may be that it established same number of transcription complexes comparing with other vectors (for example, pET-AluY × 1, carrying 1 copy of AluY) so that pET-AluY × 14 vector (carrying 14 copies of AluYs) produced more AluY RNAs than that of pET-AluY × 1 vector.

### The effects of types of host bacteria and culturing temperature of the bacteria on production amount of AluY RNAs

**Fig. 3:**
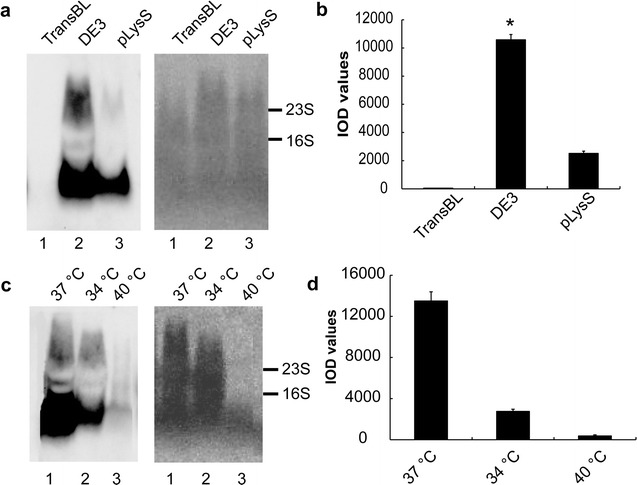
The effects of types of host bacteria and culturing temperature of the bacteria on yield of AluY RNAs. **a** Effects of types of BL21 host bacteria on yield of AluY RNAs. The left panel shows the result of northern detection. pET-AluY × 8 plasmid was transformed into three kinds of BL21 cells, including DE3, *BM*BL21-DE3-pLysS (pLysS) and Trans BL 21 (TransBL), northern blotting showed that the yield of AluY RNAs was the highest when using pET-AluY × 8 DE3; the right panel shows the dyeing results of methylene blue; **b** IOD values of each lane of the northern blotting results (means of three independent experiments) of types of BL21 host bacteria on yield of AluY RNAs. “*”, the AluY RNAs yield was the highest using DE3 engineering bacteria and was significantly higher than that of other bacteria (p < 0.01). **c** Effects of culturing temperature of pET-AluY × 8 DE3 bacteria on yield of AluY RNAs. The left panel shows the results of northern blotting. pET-AluY × 8 DE3 bacteria were cultured at 34, 37 or 40 °C and then were induced by IPTG for 4 h, we found AluY RNAs yield was the highest under the 37 °C cultivation; the right panel shows the dyeing result of methylene blue; **d** IOD values of each lane of the northern blotting results of effects of culturing temperature (means of three independent experiments). “*”, the AluY RNA yield was the highest under the 37 °C cultivation and was significantly higher than that of other temperature (p < 0.01)

Three different strains of BL21 *E. coli* including *BM*BL21-DE3 (DE3), *BM*BL21-DE3-pLysS (pLysS) and Trans BL 21 (TransBL) were used as host bacteria. When multiple tandem AluY sequences (for example 14 copy of AluYs) were inserted into pET, the inserted AluY sequences sometime recombined and lead to change of copy number of AluYs. For the stability of the experimental results, we used pET-AluY × 8 vector to explore the optimal conditions of producing AluY RNAs. pET-AluY × 8 TransBL, pET-AluY × 8 DE3 and pET-AluY × 8 pLysS bacteria were cultured to OD_600_ reached 1.0 respectively, then induced for 4 h at 37 °C using IPTG at final concentration 0.2 mg/ml. AluY RNAs was extracted and assessed using northern blotting shown in Fig. 3a. The right panel of Fig. 3a shows the loading amount in each lane dyed using methylene blue. Figure 3b shows the IOD values of northern blotting results. The results of northern blotting showed that the yield of AluY RNAs were the highest when using DE3 bacteria. pLysS bacteria are suitable to produce toxicity engineered protein from pET derived plasmids; TransBL bacteria are not suitable to produce engineered protein from pET derived plasmids; DE3 bacteria are suitable to produce nontoxicity engineered protein from pET derived plasmids. Our results showed that DE3 bacteria were suitable to produce AluY RNAs.

Next, pET-AluY × 8 DE3 bacteria were cultured to OD_600_ 1.0, then induced for 4 h at different temperature (34, 37 or 40 °C) with IPTG induction (final concentration 0.2 mg/ml). AluY RNAs was extracted and assessed using northern blotting shown in Fig. 3c. The right panel of Fig. 3c shows the loading amount in each lane dyed using methylene blue. Figure 3d shows the IOD values of northern blotting analyzed by Gel-Pro-analyzer software. The results of Fig. 3c, d showed that the yield of AluY RNAs was the highest under the 37 °C cultivation and was significantly higher than that of other culture temperature (34 and 40 °C). It has been reported that yield of hIL-2-mGM-CSF protein increased with increasing of culture temperature and the yield was the highest under the 42 °C cultivation [21]. However, our results showed that yield of AluY RNAs under the 37 °C cultivation was the significantly higher than that of 34 °C or 40 °C culture temperature. The reason that increasing temperature did not increase the yield of AluY RNAs may be that AluY RNA degradation was high or its production was less under 40 °C cultivation.

### Effects of digestion of DNA enzyme and RNA enzyme on AluY RNAs yield

**Fig. 4:**
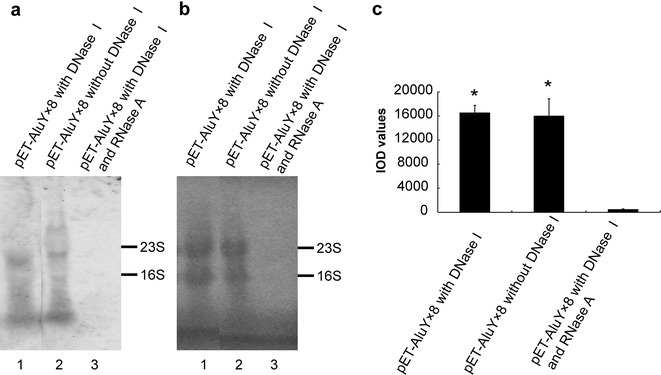
Effects of digestion of DNA enzyme and RNA enzyme. **a** The result of northern detection. pET-AluY × 8 DE3 bacteria were induced with IPTG (final concentration 0.2 mg/ml) at 37 °C for 4 h. RNAs were extracted using SDS-hot phenol method and digested with DNase I (lane 1), without DNase I (lane 2), or with DNase I plus RNase A (lane 3). AluY RNAs were detected using northern blotting. **b** Dyeing results of methylene blue; **c** IOD values of each lane of the northern blotting results (means of three independent experiments). “*”, the AluY RNA yield of pET-AluY × 8 DNase I digestion and pET-AluY × 8 without DNase I digestion was significantly higher than that of pET-AluY × 8 DNase I plus RNase A digestion (p < 0.01)

RNAs being extracted using SDS-hot phenol method [11] from pET-AluY × 8 DE3 bacteria were digested with DNase I (Fig. 4a, lane 1), without DNase I (Fig. 4a, lane 2), or with DNase I plus RNase A (Fig. 4a, lane 3). AluY RNAs were assessed by northern blotting. The hybridization signal in lane 1 and lane 2 of Fig. 4a was similar, illustrating that plasmid DNAs did not play key roles in producing the hybridization signals. There is no the hybridization signal in lane 3 of Fig. 4a, suggesting that RNA played important roles in producing the hybridization signals. Figure 4b showed that dyeing extent in lane 1 and line 2 was similar, suggesting that DNase I digestion did not affect the amount of RNAs. Line 3 in Fig. 4b was not stained, suggesting that RNase A degraded all RNAs. Figure 4c shows the IOD values of each lane of the northern blotting results (means of three independent experiments). The AluY RNA amount of pET-AluY × 8 DNase I digestion and pET-AluY × 8 without DNase I digestion was significantly higher than that of pET-AluY × 8 DNase I plus RNase A digestion (p < 0.01).

### The estimation of yield of pure AluY RNAs

**Fig. 5:**
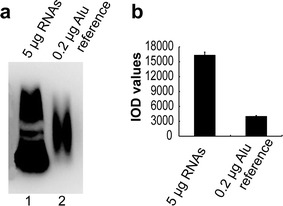
The estimation of yield of pure AluY RNAs. **a** The results of northern detection. RNAs were extracted from pET-AluY × 14 DE3 bacteria induced by IPTG and digested with DNase I. 5 μg RNAs digested with DNase I were loaded in lane 1, 0.2 μg Alu reference was loaded in lane 2; **b** IOD values of each lane of the northern blotting results (means of three independent experiments)

Because AluY RNA yield from pET-AluY × 14 DE3 bacteria was the highest (Fig. 2a, lane 5), pET-AluY × 14 DE3 bacteria were used to perform the following experiments of the estimation of yield of pure AluY RNAs. pET-AluY × 14 DE3 bacteria were cultured to 1.0 OD_600_, induced by 0.2 mg/ml IPTG for 4 h at 37 °C. RNAs (total RNAs) were extracted and yield of RNAs was measured. Mean value of RNA yield of 100 ml bacteria solution in six independent experiments was 2.94 ± 0.59 mg. AluYRNAs were assessed by northern blotting, meanwhile sense AluY single stranded DNA as reference substance (Alu reference). 5 μg RNAs digested with DNase I were loaded in lane 1 of Fig. 5a, 0.2 μg Alu reference was loaded in lane 2 of Fig. 5a; Fig. 5b shows the IOD values of each lane of the northern blotting results (means of three independent experiments). The hybridization signal strength of lane 1 (loading with RNAs) was 3.95-fold of that of lane 2 (loading with Alu reference DNA). The pure AluY RNAs occupied 15.8% of RNAs if ignoring the difference of the binding ability of DNA and RNA. The computational process of ratio of pure AluY RNAs in RNAs as below: the amount of Alu reference (0.2 μg) × (IOD value of RNA lane/IOD value of Alu reference) = amount of pure AluY RNAs (μg); the amount of pure AluY RNAs/the loading amount of RNAs × 100% = the ratio of pure AluY RNAs. Each 100 ml bacteria solution produced 2.94 mg RNAs and pure AluY RNAs occupied 15.8%, thus each 100 ml bacteria solution can produce 0.46 mg pure AluY RNAs.

## Discussion

RNAs play important roles in regulating gene expression [22–25]. Elbashir et al. [26] proved that synthetic siRNA transfected into the cultured mammalian cells degraded target mRNA and inhibited gene expression after entering RNA inducing silencing complex. Our recent works found that synthetic RNAs (23 nt) complementary to mouse albumin gene and total mouse liver RNA increased DNase I digestion sensitivity of mouse albumin gene, suggesting that RNAs can increase chromatin accessibility [11]. At present, the methods of acquiring extracellular RNAs include RNA synthesis using RNA synthesizer [9], in vitro transcription [10] and extraction from cells [11]. BL21 *E. coli* are defective bacteria and the common bacteria for producing genetic engineering proteins and can produce many types of genetic engineering proteins after appropriate plasmids transformation and IPTG induction [27, 28]. In this paper, the preparation technology of genetically engineered humanized AluY RNAs was established using BL21 *E. coli*. The RNAs prepared using this method can be used for biology experiments.

pET-AluY × 8 DE3, pET DE3 and DE3 were cultured using Luria–Bertani (LB)-kanamycin medium, respectively. The growth of all types of bacteria reached plateau at OD 2.6 without IPTG induction. The growth curve of DE3 bacteria with IPTG induction was similar with that of without IPTG induction (Fig. 1a); the growth of pET-AluY × 8 DE3 and pET DE3 bacteria reached plateau at OD 1.3 with IPTG induction, which illustrates that IPTG induction inhibited the growth of the two types of bacteria. These results illustrated that IPTG induction did not affect the growth of DE3 bacteria, while combination of IPTG and pET plasmid reduced the growth of DE3 bacteria. Both transformation of pET and pET-AluY × 8 plasmids reduced the growth of the bacteria with IPTG induction, which illustrates that the reason of inhibiting the growth of the bacteria was from pET plasmid rather than AluY insertion.

Shafiee et al. [29] showed the highest level of DT386-BR2 fusion protein expression was attained after 2 h of incubation with IPTG and increasing the incubation time did not affect the amount of expression; in this paper, we found that AluY RNA yield reached peak at 4 h of IPTG induction, remarkably decreased after 8 h with IPTG induction. The reason of inducing AluY RNA yield decreased maybe that RNA easily degrade. Shafiee et al. [29] found that the IPTG concentration had the most effect on protein expression and the optimal IPTG concentration was 1 mmol/l; in this paper, we found that optimal IPTG concentration was 0.1–0.4 mg/ml (0.42–1.68 mmol/l) (Fig. 1d). The role of IPTG induction is to activate *lac* operon system and induce expression of the gene encoding the T7 RNA polymerase (T7 RNAP). The T7 RNAP specifically recognizes the T7 promoter that drives the transcription of the AluYs inserted downstream of T7 promoter [30].

The unique design in this paper is that the AluY RNA yield increased with increasing the copy number of AluY elements and expression amount of AluY RNAs in pET-AluY × 14-DE3 was twofold of that in pET-AluY × 8-DE3 (Fig. 2a). However, this method is not suitable to increase yield of genetic engineering protein since producing genetic engineering protein need to consider gene reading frame and structure of non-coding regions at 5ʹ upstream and 3ʹ downstream.

In this paper, three different strains of BL21 *E. coli* were used as host bacteria. DE3 bacteria are suitable to produce nontoxicity engineered protein from pET derived plasmids; pLysS bacteria are suitable to produce toxicity engineered protein from pET derived plasmids; TransBL bacteria are not suitable to produce engineered protein from pET derived plasmids. In this paper, we found that both pET-AluY × 8 DE3 and pET-AluY × 8 pLysS bacteria produced AluY RNAs with IPTG induction and pET-AluY × 8 DE3 bacteria had the higher yield (Fig. 3a, b), while pET-AluY × 8 TransBL bacteria did not produce AluY RNAs with IPTG induction (Fig. 3a, lane 1). These results suggest that appropriate host bacteria are important for inducing AluY RNA production. pET-AluY × 8 DE3 bacteria did not produce AluY RNAs without IPTG induction (Fig. 1d, lane 1), suggesting IPTG induction is necessary for AluY RNA production. The transcription of inserted sequences in pET plasmids needs T7 RNA polymerase that belongs to IPTG inducible polymerase. DE3 and pLysS bacteria contain IPTG inducible T7 RNA polymerase, while TransBL bacteria do not contain this type of polymerase. pET-AluY × 8 DE3 bacteria could neither produce T7 RNA polymerase nor AluY RNAs without IPTG induction and pET-AluY × 8 TransBL bacteria did not produce AluY RNAs with IPTG induction, which illustrates that AluY RNAs induction had specificity. The results of engineering bacteria producing AluY RNAs under IPTG induction are consistent with that of inducing engineered proteins [31].

To detect the roles of DNAs and RNAs in northern hybridization signal, we performed nuclease digestion experiments. The hybridization signal intensity of digestion with DNase I was similar with that of without DNase I digestion (Fig. 4a, lane 1 vs lane 2), illustrating that DNA contamination did not play important role on the hybridization signal intensity; the hybridization signal totally disappear after digestion with RNase A plus DNase I digestion (Fig. 4a, lane 3), illustrating that the hybridization signal belong to AluYRNAs.

In this paper, we adopted SDS-hot phenol method to extract RNAs so that most of AluY RNAs are short fragments. The size of AluY RNAs was ascertained approximately 600 nt long using Gel-Pro-analyzer software, meanwhile, 23S rRNA (2900 nt) and16S rRNA (1540 nt) were used as molecular weight marker. The results of methylene blue dye on northern blotting membranes showed that the ratio of 23S rRNA and 16S rRNA was approximately 2:1 (Fig. 1b, the bottom panel), illustrating that RNAs extracted using SDS-hot phenol method did not degrade. Thus, the reason that most of AluY RNAs are short fragment is not due to the degradation during RNA extraction process.

In this paper, we performed quantitative determination of AluY RNAs using AluY single stranded DNA as reference substance. If the hybridization signal difference of DNA single strand and RNA single strand with probes can be ignored, hybridization signal difference of DNAs can reflect the that of RNAs. The loading amount of RNAs in a lane was 5 μg, the amount of single stranded AluY DNA in a lane was 0.2 μg. The hybridization signal strength of AluYRNAs was 3.95-fold that of Alu reference (Fig. 5), so 5 μg loading RNAs contained 0.79 μg pure AluY RNAs, so pure AluY RNAs occupied 15.8% of RNAs. The first main ingredient of bacteria total RNA is rRNA and then is tRNA. In this paper, T7 promoter and IPTG induction were employed, pure AluY RNAs occupied 15.8% of RNAs, 100 ml bacteria solution can produce 0.46 mg pure AluY RNAs.

## Conclusions

In this paper, we established the technological methods of preparing genetically engineered humanized AluY RNAs (AluY RNAs) from *E. coli* strains. This technology also can be used to prepare other genetically engineered humanized RNAs that can be used for cytology experiments. The optimal conditions of producing AluY RNAs were an engineering bacteria concentration of OD_600_ 1.0 (DE3 as host bacteria), a tandem AluY number of 14 copies, an IPTG concentration of 0.2 mg/ml, a culturing temperature of 37 °C and a culturing time of 4 h. Pure AluY RNAs occupied 15.8% of extractive total RNAs and the mean yield of pure AluY RNAs in 100 ml bacteria solution was 0.46 mg. Further improving the purity of AluY RNAs using affinity chromatography technology will be next study plan.

## Methods

### Construction of pET-AluY plasmids

**Fig. 6:**
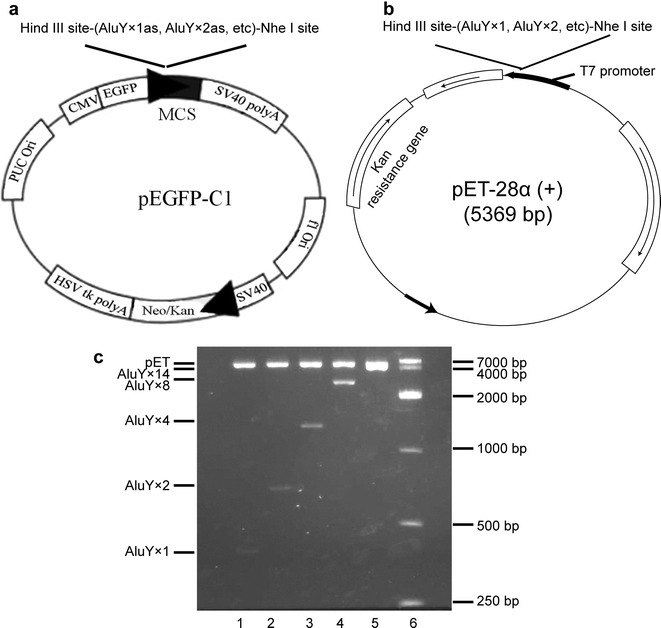
The diagrammatic sketch of plasmid construction and enzyme digestion. **a** Schematic diagram of pEGFP-C1 derived vectors. The tandem antisense AluY sequences (AluY × 1as, AluY × 2as AluY × 4as AluY × 8as AluY × 14as) were inserted the multiple cloning site (MCS) of pEGFP-C1 vector. The order of restriction enzyme cutting sites in pEGFP-C1 vector was 5′-*Hin*dIII site-antisense Alus-*Nhe*I site-3′, the arrows show the orientation of transcription. **b** Schematic diagram of pET derived vectors. Tandem antisense AluY sequences (relative to pEGFP-C1) digested from pEGFP-C1 derived vectors (C1-AluY × 1as, C1-AluY × 2as, C1-AluY × 4as C1-AluY × 8as, C1-AluY × 14as) were inserted into pET. *Nhe*I site in pET vector is located downstream of T7 promoter so that antisense AluYs in pEGFP-C1 inserted into pET vector transcribed sense AluY RNAs. The order of restriction enzyme cutting sites in pET vector was 5′-T7 promoter-*Nhe*I site-Alus-*Hin*dIII site-3′. **c** Agarose gel electrophoresis images of plasmids being digested with *Hin*dIII/*Nhe*I (ethidium bromide staining). Lane 1: pET-AluY × 1, lane 2: pET-AluY × 2, lane 3: pET-AluY × 4, lane 4: pET-AluY × 8, lane 5: pET-AluY × 14, lane 6: marker

Human whole AluY sequence (283 bp long, RP11-29107 clone) includes two fragments (FLAM and FRAM) joined by an A-polymeric sequence. The AluY sequence was amplified by PCR method using RP11-29107 clone as template. The primers for AluY were: forward: 5′-ATC GGA ATT CTT AAT CTA GAT AAG GCT GGG CGC GGT GGC TCA C-3′, reverse: 5′-ATC GGG TAC CAT GCT AGC TGA GAC GGA GTC TCG CTG TG-3′ [32]. The tandem antisense AluY sequences (AluY × 1as, AluY × 2as AluY × 4as AluY × 8as AluY × 14as) were inserted the multiple cloning site (MCS) of pEGFP-C1 vector (C1) to construct the plasmids including C1-AluY × 1as, C1-AluY × 2as, C1-AluY × 4as, C1-AluY × 8as and C1-AluY × 14as, have been constructed in our previous study (Fig. 6a) [32]. The order of restriction enzyme cutting sites in pEGFP-C1 vector was 5′-*Hin*dIII site-antisense Alus-*Nhe*I site-3′, the arrows in Fig. 6a show the orientation of transcription. These pEGFP-C1 derived plasmids were digested separately by *Hin*dIII/*Nhe*I, were subjected to 1% agarose gel electrophoresis. The inserted fragments were separated. pET plasmid was digested by *Hin*dIII/*Nhe*I, subjected to 1% agarose gel electrophoresis, cut plasmid band to separate pET plasmid DNA from gel. The inserted DNA fragments and pET plasmid DNA were ligated using T4 DNA ligase to construct pET-AluY × 1, pET-AluY × 2, pET-AluY × 4, pET-AluY × 8 and pET-AluY × 14 plasmids (Fig. 6b). After tandem antisense AluY sequences (relative to pEGFP-C1) were inserted into pET, *Nhe*I site in pET vector is located downstream of T7 promoter so that antisense Alus inserted into pET transcribed sense Alu RNAs. The order of restriction enzyme cutting sites in pET vector was 5′-T7 promoter-*Nhe*I site-Alus-*Hin*dIII site-3′. Figure 6c shows agarose gel electrophoresis images of plasmids being digested. The size of fragments meet expectation.

### BL21 *E. coli* transformation and IPTG induction

Three types of BL21 *E. coli* cells, including DE3,pLysS and TransBL, were used in this study. These competent cells were transformed by pET or pET-AluY plasmids using thermal stimulation method, smear on LB-kanamycin agar plates. The plasmid DNA was extracted, digested and electrophoresed to select bacterial colonies with the correct plasmids. Then these BL21 bacteria transformed by the plasmids with correct plasmids were cultured to OD_600_ 1.0, and then induced with IPTG for a period of time.

### The preparation of AluY RNAs

RNAs were extracted using SDS-hot phenol method as described elsewhere [11]. Briefly, after 10 ml bacteria liquid was centrifuged, the precipitate was treated using 2 ml 2% SDS-0.15 mol NaCI, added 1 ml water phenol for 30 min at 60 °C, added 0.5 ml chloroform after cooling. The liquid supernatant was collected after centrifugation, precipitated with threefold volume absolute ethyl alcohol for 30 min at 4 °C, centrifuged for 10 min at 12,000 rpm. The precipitate was washed three times with 75% ethanol to gain RNAs. DNase I (TaKaRa Biotechnology, Japan) and RNase inhibitor (Thermo Scientific, USA) were added at a final concentration of 0.5 U/ml to wipe off contaminating DNAs. Then RNAs without contaminated DNAs were used to detect AluY RNAs using northern blotting method.

### AluY RNA amount was detected using northern blotting

To prepare 0.25 mmol/l biotin-dNTP, dATP (1 mmol/l) 75 μl, dCTP (1 mmol/l) 75 μl, dGTP (1 mmol/l) 75 μl, dTTP (1 mmol/l) 50 μl and biotin-16-dUTP (1 mmol/l, Roche biotech company, Switzerland) 25 μl were mixed. To obtain biotin labeled AluY probe, a 140 bp fragment from AluY sequence was amplified by PCR using C1-AluY × 8 plasmid as template, AluY140F as forward primer (5ʹ-GTGGTGGCGGGTGCCTGTAG-3ʹ) and AluYR (5ʹ-TGAGACGGAGTCTCGCTGTG-3ʹ) as reverse primer, the PCR reaction mixture contained biotin-dNTP with a final concentration of 0.1 mmol/l and other conventional components. PCR was performed using the following conditions: 94 °C for 30 s, 50 °C for 30 s, and 72 °C for 1 min for 30 cycles. Although preparative 140 bp long AluY probe targeting to right arm of the AluY, the probe can detect the whole AluY sequence, since right arm is located in downstream of the whole AluY sequences and the transcription of AluY is from its upstream.

RNAs were electrophoresed on a 1.2% agarose gel containing 0.4 M formaldehyde, and then transferred to nylon membranes (pore diameter = 0.45 μm; Osmonics, USA) [22]. The nylon membranes were dyed using methylene blue, blocked with preliminary hybrid liquid (1% skim milk powder—0.1 mg % salmon sperm DNA), and then hybridized with biotin-labelled AluY probes at 42 °C for overnight in a UL2000 hybriLinker (UVP, USA).

The membranes were washed twice using a solution of 1 × SSC − 0.1% SDS at room temperature, reacted with 1:200 diluted avidin-peroxidase (Boster Biological Technology, Wuhan, China) for 1 h, washed for three times, and then reacted with the horseradiase chemiluminescent fluid (Novex Life Technologies, USA). The imaging analysis system (ChemiDocTM Touch, Bio-RAD) was used to analyze the results and Gel-Pro-analyzer software was used to analyze the IOD values.

## Abbreviations

IPTG: isopropyl-β-d-thiogalactoside
IOD: integral optical density
LB: Luria–Bertani
*E. coli*: *Escherichia coli*
DE3: *BM*BL21-DE3
pLysS: *BM*BL21-DE3-pLysS
TransBL: Trans BL21
C1: pEGFP-C1
pET: pET-28α
